# “Burying the proud part of my body”: a Chinese student-athlete's autoethnography from concussion recovery to role exit

**DOI:** 10.3389/fspor.2026.1736997

**Published:** 2026-08-31

**Authors:** Zichen Zhuang, Hongjiang Wang

**Affiliations:** College of Physical Education, Hangzhou Normal University, Hangzhou, Zhejiang, China

**Keywords:** American football, autoethnography, China, role exit, social-ecological theory, sport-related concussion, student-athletes

## Abstract

Student-athletes often struggle to maintain the demands of a dual career in academics and sports. A severe injury can trigger role exit; when the injury is a sport-related concussion (SRC), challenges extend beyond biomedicine—recovery is shaped by social interactions and sociocultural norms and may threaten the physical, psychological, and academic functioning of student-athletes. Despite the growing number of individuals participating in sports, China has limited qualitative research centered on lived-in SRC experiences. Drawing on an autoethnographic account of SRC-triggered role exit and guided by social-ecological theory, this study examines which multi-level social factors generate anxiety, hinder rational decision-making, and re-emerge after SRC and which factors buffer recovery and support exit. The analysis shows that scarce resources and the marginalization of non-system sports participation can intensify personal overinvestment and rapidly centralize athlete identity; this identity both sustains persistence and later impedes transition, amplifying catastrophic thinking, self-doubt, and reluctance during exit. While academic demands heighten dual-track strain, academic capital and attainable goals can become key resources for self-managed recovery and for post-exit identity reconstruction by converting role residuals into research and career advantages. Interpersonally, teammates' instrumental and esteem support, non-moralizing teacher/supervisor support, and family companionship provide critical buffering. At the meso and macro levels, however, limited medical communication, the absence of executable return-to-learn/return-to-play procedures, and the lack of coordinated policy support leave recovery privatized and uncertain, complicating both rehabilitation and decision-making after SRC.

## 1 Introduction

In 2020, former Pro Bowl player Luke Kuechly made the unexpected decision to retire at what appeared to be the peak of his career. I (the author ZZ) still remember him, tears in his eyes, saying: “I still want to play, but I just feel like it isn't the right decision” (1, 2). Three years later, after being driven hard to the ground during a red-zone defensive play and experiencing amnesia, I realized that the difficult choice I had once only seen in the news was now standing squarely in front of me.

Although the stage of student-athletes is far less dazzling than that of professionals, when injury occurs, we face the same fundamental question: is it worth continuing an athletic career at the risk of long-term health impairment and academic disruption (3)? In those moments, anxiety and catastrophic thinking are often markedly amplified (4, 5). Even when one manages to leave sports “successfully,” long-term psychological distress may persist (6). This is especially true when the injury is a sport-related concussion (SRC), whose complexity far exceeds that of many visible injuries (7). SRC is not only a biomedical issue (8); it is also profoundly shaped by psychological factors and by external environments such as family dynamics, school contexts, social norms, and institutional support, which continually influence how individuals interpret symptoms and how recovery unfolds (9, 10). Accordingly, researchers have called for greater attention to the lived-in experiences of those with SRC, and this topic has been widely discussed internationally (11–14).

In China, however, attention to SRC remains limited. Although official statistics indicate that, as of 2024, the proportion of the population who regularly participate in physical exercise has exceeded 38.5% (15), domestic concern for SRC is still scarce. Both the general population and educators show an overall lack of understanding of SRC (16, 17). Moreover, research in China not only lacks in-depth investigation of SRC but also rarely adopts qualitative approaches centered on the perspectives of individuals who have personally experienced SRC. As a result, within the Chinese sociocultural context, the profound psychological and social experiences of SRC survivors are highly likely to be overlooked.

Against this backdrop, as a former student-athlete who experienced a concussion in the Chinese social environment, struggled to balance academics and competition, and ultimately developed a role-exit (18) narrative, I possess an intense embodied experience. This study uses an autoethnographic approach (19) to reflectively write about my personal narrative, connecting individual experience to broader networks of social interaction (20). By supplementing accounts of SRC experiences situated in non-Western cultures and institutional settings, this study aims to provide a concrete point of reference for Chinese student-athletes facing similar dilemmas and to offer empirical support for building a health support system for student-athletes that aligns with China's national conditions. Inspired by social ecological theory (21), this study addresses the following questions:

1. In the Chinese context, which prior experiences and multi-level social factors constitute sources of anxiety for student-athletes, hinder rational decision-making, and re-emerge when SRC occurs?
2. During SRC recovery and the subsequent process of “role exit,” which multi-level social factors and individual resources function as buffers and sources of support?

## 2 Literature review

### 2.1 SRC

SRC is a type of traumatic brain injury caused by a direct blow to the head, neck, or body, through which external force is transmitted to the brain (22). This injury occurs during sports- or exercise-related activities. Although routine structural imaging is often normal, symptoms such as headaches, dizziness, balance impairment, and amnesia may occur (23, 24). Beyond American football, sports such as men's and women's soccer, basketball, and track and field also involve a nontrivial risk of concussion (25, 26).

Compared with the majority of other sports injuries, concussions carry a higher risk of subsequent psychological problems (27). For students, concussion-related cognitive fatigue and attentional limitations may also increase subjective academic difficulty, making school-level accommodations necessary, such as reduced coursework, extended exam time, and reduced screen exposure (28). Because concussions are largely invisible, their identification and diagnosis in practice depend heavily on individuals' disclosure of subjective symptoms and on recognition by observers (29). This underscores the need to study the experiences and perspectives of individuals who sustain a concussion. Prior research has shown that individuals often underreport, “tough it out,” or delay reporting (30, 31) due to motivations such as wanting to continue playing, not wanting to miss schoolwork (29), or pressures stemming from team expectations, traditional masculine “toughness” norms, and pain culture (32). Beyond physiological indicators, the psychological and social dimensions of concussions have a stronger influence than many visible injuries do, which calls for more qualitative work to deepen understanding (12).

Within the Chinese context, SRC has a relatively low level of public problematization and institutionalization. Due to limited individual awareness, insufficient attention to documentation, and a lack of case identification and traceable samples, the empirical foundation for research on sport-related concussion remains weak (33–35). Existing work is largely restricted to surveys of concussion knowledge (33), localization of assessment tools (34), or reviews and recommendations (16, 36). Studies based on large samples of injured individuals or on in-depth accounts of lived-in experience remain scarce.

### 2.2 Student-athletes

Student-athletes typically refer to individuals who simultaneously and continuously invest in both academic and athletic tracks, maintain academic standards to preserve athletic eligibility, and comply with an amateur athletic status, meaning they may not receive compensation for competition (37, 38). Some elite athletes receive substantial support through stipends or Name, Image, and Likeness (NIL) arrangements (39, 40), or benefit from career safeguards embedded in institutional systems; these athletes tend to commit more decisively to athletic competition and experience less subjective pressure to balance academics (41). However, the majority of student-athletes continue to face pressures arising from time conflicts, competing demands on physical and mental energy, and limited economic and institutional resources, and therefore must struggle to maintain a dynamic balance between academics and sports (37, 38, 42–44).

A parallel pattern exists in China. At the macro level, within a planned development environment oriented toward elite performance (45), student-athletes also display strong dependence on institutions. Those within the system receive institutionalized support—such as recruitment and management by elite teams, grade-to-credit conversion mechanisms, and sport-specific bonuses—thereby gaining clear advantages in resources and opportunities (46, 47). In contrast, the majority of student-athletes exist outside the formally established system (47). In practice, they are often the ones who truly embody the government's advocated “integration of sports and education,” bearing the dual burden of study and training. They also shoulder pressure from academics, training, competition, and daily life, pressures that are directly linked to stress responses and mental health (48). However, when accessing resources, obtaining identity recognition, and receiving support services, they must pay higher costs and face greater uncertainty (46, 49). University student-athletes have become one of the groups at the highest risk for mental health problems in China, with factors such as depression, hostility, and phobic anxiety showing sustained deterioration over a 28-year period (50).

### 2.3 Role exit

Sports are not without risk. Despite precautions, injuries will sometimes happen (51). Participation in sports makes injury difficult to avoid. When an injury is severe, recurrent, involves prolonged rehabilitation, or entails unfavorable long-term health risks (52), it can become a trigger that—under the cumulative influence of social, school, and institutional factors—prompts student-athletes to consider retirement, a process that often entails struggle (5, 53, 54). Particularly during rehabilitation, they may fall into anxiety and ambivalence (55). If they choose to retire, they must also settle their minds and engage in self-adjustment (56, 57), accepting that their identity as athletes has become part of the past.

The concept of role exit (18) captures the process through which college athletes transition out of the competitive role, strip away their identity as athletes, and place it into their personal history. Role exit has been defined as a full process—from the initial emergence of intent, through the final decision, to post-exit identity reconstruction (58). It encompasses both repeated deliberation over whether to exit and the subsequent challenge of adapting and re-adjusting after exit (18). It has been used to explain the stage-based social process and identity reconstruction involved when adolescents and college athletes withdraw from sports (58, 59).

Decisions throughout the role-exit process are shaped not only by the individual but also by influences from society, schools, and institutions (40, 59–61). Therefore, this study draws on social ecological theory (62) to discuss the broad influences on student-athletes within a multilevel and operational analytical framework. This theory posits that individual behavior and development result from interactions with the surrounding social environment, ranging from immediate micro-level settings to broader cultural contexts. At each level—individual psychology (63), interpersonal relationships (64), environmental institutions, and cultural norms (65)—factors play important roles from the personal to the macro level.

## 3 Methods

This study employs an autoethnographic approach, combining personal narrative with ethnographic observation and positioning the researcher as both the subject and the object of the inquiry (19). Its core features include reflexivity, authenticity, self-awareness, narrative, and sensitivity to context (66). These features enable the method to reveal the “invisible impacts” of an invisible injury, such as a concussion. For example, Dean (67) used autoethnography to document their experience of a sport-related concussion: appearing normal on the surface and continuing to play, while in fact enduring physical pain and emotional distress, followed by a breakdown associated with role exit and, ultimately, self-adjustment and identity reconstruction (67, 68). At the same time, autoethnography allows us to see institutional and cultural norms through individual narratives, for instance, revealing the normalization of pain in collision sports and the disciplining force of gender norms, both of which shape the choices and identity renegotiation of injured athletes (69, 70). This approach is therefore well-suited to the present study.

Nevertheless, autoethnography is often criticized for drifting into overly self-indulgent or narcissistic writing (71), becoming overly immersed in the personal story while lacking reflection on broader social phenomena (20), and risking distortion of materials under romanticized, scene-driven narrative styles (72).

In response, first, self-indulgence and narcissism imply deriving gratification from the process (67). However, I acknowledge that, during writing, I continue to feel shame about exposing private experiences and inner vulnerability (73) and am unconsciously inclined to avoid such exposure; for me, this provides no sense of gratification. Instead, my motivation stems from the hope that more individuals will address the structural dilemmas faced by athletic populations such as mine, and by the desire to provide experiential reference and resonant support for other student-athletes. Second, this study explicitly adopts social ecological theory; throughout the text, narrative episodes are tightly interwoven with dialogue from the literature, moving beyond the personal to link subjective experience to macro-level discursive debates. The intention is to use my role-exit narrative as a pathway toward deeper explanations at the interpersonal, meso-, and macro-levels of institutions and culture (71, 74).

Finally, expanding the sources of memory is a key strategy for enhancing the quality of ethnography (71). To avoid relying solely on self-report and memory, I triangulated multiple materials, including diaries, communication texts, and visual records, along with key-informant interviews with relevant individuals. These sources were compared and integrated to ensure credibility and to establish triangulation (75).

### 3.1 Self-as-participant

Because this study adopts autoethnography, the first author (referred to as “I”) is also the focal participant (Table 1). The narrative spans experiences from 2019 to 2024. Since 2016, I have been an American football fan, but had no opportunity to participate. In 2018, I studied Software Engineering at University A in Hangzhou. In 2019, I joined a club and began training in American football. In 2022, I progressed to University B. In 2023, I played in China's largest American football league, the CNFL, serving as the starting safety. During the 2024 preseason, I experienced the SRC event described in this narrative and developed amnesia. After the 2024 season ended, I retired.

**Table 1:** Researcher positionality details.

Item	Details
Pronouns	He/Him
Age (at the time of the study)	25
Nationality	Chinese
Education	2018–2022: Bachelor's in Software Engineering
	2022–2025: Master's in Sport Humanities and Sociology
Institutions (location)	Undergraduate: Hangzhou A University
	Master's: Hangzhou B University
Sport Involvement	2019–2021: Hangzhou American Football Club A
	2022–2024: Hangzhou American Football Club B
Training Load	Conditioning: 3–5 sessions per week (varying by off-season/in-season/post-season)
	Team A: training once per week; distance 60 km
	Team B: training twice per week; distance 30 km
League Experience/Position	2024 CNFL—Starting Free Safety
	2023 CNFL—Starting Free Safety

### 3.2 Data collection

Autoethnography encourages researchers to organize time-relevant materials from the past as “prompting cues” for memory elicitation and narrative reconstruction (76, 77).

Since I proposed my master's thesis in 2022—an observational study of community participation among Chinese American football players (78)—I maintained relatively systematic field diaries during my core participation in the CNFL season, from August 2023 to November 2024.

For earlier periods, including memories from my initial involvement in American football training during my undergraduate years, I also incorporated my social media posts, informal diaries, and communication texts as qualitative data (79). Team A and Team B publicly release game videos and photographs for each match. These visual materials served as memory cues (80) and were also used to organize and retrospectively consolidate materials.

In addition, we employed key informant interviews (81), interviewing one coach, one team administrator, two teammates, and one teacher. These interviews supplemented and further clarified the events I experienced. Together with diaries, communication texts, and visual materials, these sources formed triangulation across multiple data streams.

Notably, I experienced at least six hours of amnesia around the concussion and several days of memory disturbance (82). I therefore could not independently recall what happened, and some memories were unreliable. Key informant interviews, contemporaneous communication texts, and game footage were essential for reconstructing events before and after the concussion.

### 3.3 Data analysis

I used MAXQDA 24 to conduct preliminary organization of all materials, followed by narrative work.

First, I used original words, phrases, or expressions from the materials as codes and conducted an initial round of open coding. This is often used as a “coarse-grained” approach during full reading (83, 84). I then arranged all the codes and materials in chronological order. Consistent with common role-exit processes and narrative analytic practice, the narrative was organized linearly by time sequence (18, 85, 86).

Using the organized materials as prompting cues, I wrote the narrative, a practice adopted by ethnographic scholars (87). Specifically, I employed free writing (88, 89) as a route for both generating and analyzing data: each day, I wrote continuously for 10–15 min without revising or stopping. When new insights emerged, I immediately returned to the writing context, continuing until no further additions or understanding could be produced (90–92). Once the narrative's structure and meaning had cohered, I condensed the narrative and incorporated it into the manuscript, paying attention to the boundaries of theoretical contribution (93), evidence (94), and ethics (95).

To further enhance the narrative's quality and credibility, I adopted peer supervision (96) and member reflection (93) during the analysis process. Each month, I discussed the narrative with one professor of sports studies and one sports studies doctoral researcher skilled in qualitative methods to examine its credibility. These dialogues included questioning and challenges to prevent narrative closure and self-sealing. I also invited two teammates who had worked with me for more than two years and one coach to discuss the narrative. Through dialogic tension, these discussions again helped avoid the autoethnographic pitfall of self-absorption and prompted addition of contextual details, doubts, and expanded interpretations.

### 3.4 Ethical considerations

This study adheres to the Ethical Guidelines for the Social Sciences and Humanities (197), and it is overseen by a Chinese National Social Science project (project number 23BTY055). For all relevant individuals who might be drawn into the narrative, the study employed a processual consent approach (95, 198): initial consent was obtained before (re)commencing writing, and risk communication and confirmation were conducted again prior to major revisions and the final draft. In addition, specific locations, personal names, and club names appearing in this study have been anonymized in accordance with the General Data Protection Regulation (199). In the final presentation, sections involving others underwent de-identification review, and certain composite characters and reconfigured plotlines were employed to protect confidentiality while preserving narrative coherence (97, 200).

Recognizing that the risks of autoethnography pertain not only to others but also to the researcher, and that self-disclosure can expose personal vulnerabilities and trigger emotional exhaustion or psychological trauma (98), the research team also put in place supportive measures: supervisory discussions were provided by the 23BTY055 project group, and psychological support resources were made available by the School of Physical Education at Hangzhou Normal University.

## 4 Results and discussion

In autoethnographic research, scholars argue that researchers should not only present their personal experience but also work toward developing a theoretical understanding of broader social phenomena. Personal experience is used to interpret and critique context and social factors. Social ecological theory can systematically anchor “my” experience to the effects operating at the interpersonal, institutional, policy, and cultural levels, thereby pushing the narrative toward a broader and more contextualized explanation (20, 74, 99). This theory emphasizes that individual behavior or outcomes are jointly shaped by multi-level contexts (62, 100). It has been widely applied to issues such as public health, social health, rehabilitation support, and career transitions, helping to organize dispersed influences into analyzable content when explaining what factors shape a person's behavior (101–104). Researchers select or adapt the framework according to their specific contexts and research aims, rather than applying a fixed taxonomy (105–107).

**Figure 1:**
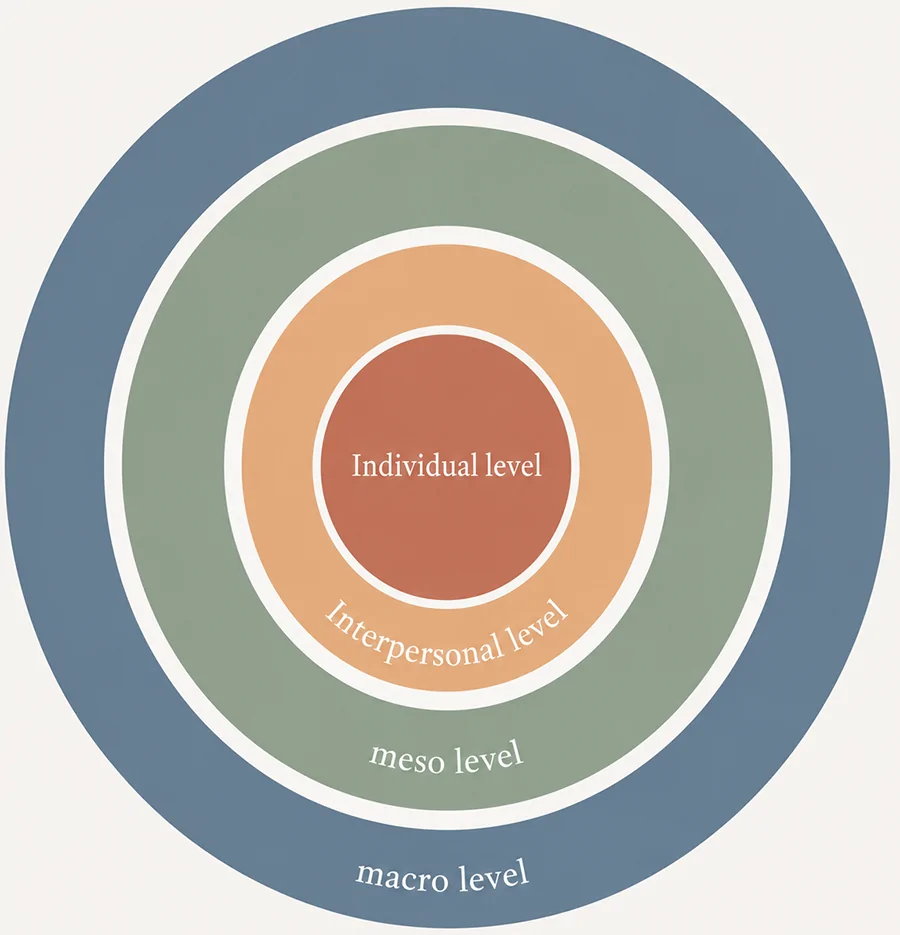
Social-ecological framework for analyzing SRC-triggered role exit.

In this article, I primarily drew on a framework used to examine social health problems (101, 102), dividing the analysis into four levels: the individual level, the interpersonal level, the meso level, and the macro level (Figure 1).

The individual level focuses on intrapersonal processes, including my psychology and cognition, identity and motivation, knowledge and skills, and health literacy. The interpersonal level focuses on close relational networks and interactions, and on influences from family members, teachers, classmates, teammates, and coaches. The meso level relates to community infrastructure, school systems, local policy implementation, and hospital practices. The macro level concerns national education, healthcare, and sports policy systems, in addition to broader sociocultural influences.

Following the principle of proximal processes, the processes operating within these levels are treated as the core engine generating developmental and behavioral outcomes. Given their relevance to my personal context, they served as triggers for discussion (62, 99). I also considered that interactions across levels are common (moderation), including upward cross-level effects. For example, meso- and macro-level conditions may not involve my direct presence, but they can indirectly shape me through factors such as resource accessibility, time pressure, informational structures, and opportunity constraints (63, 108).

### 4.1 Role entry, rapid centralization of identity, and the dual-track self

**Figure 2:**
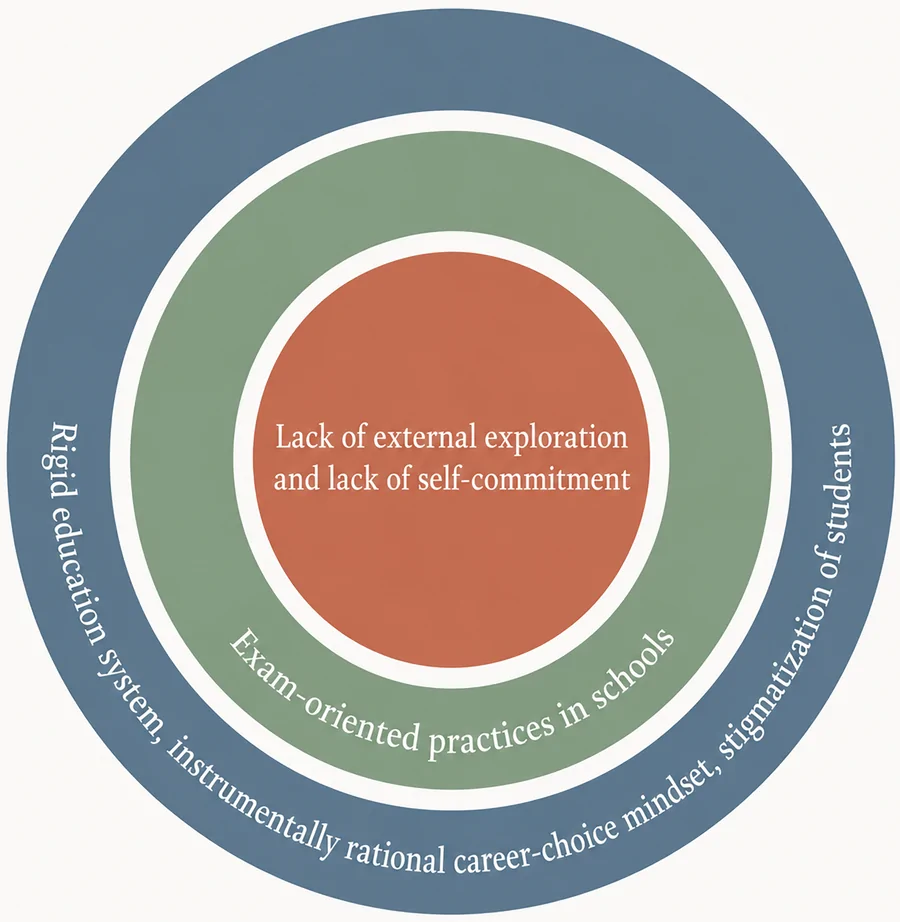
Multilevel constraints on early exploration and self-commitment.

**Figure 3:**
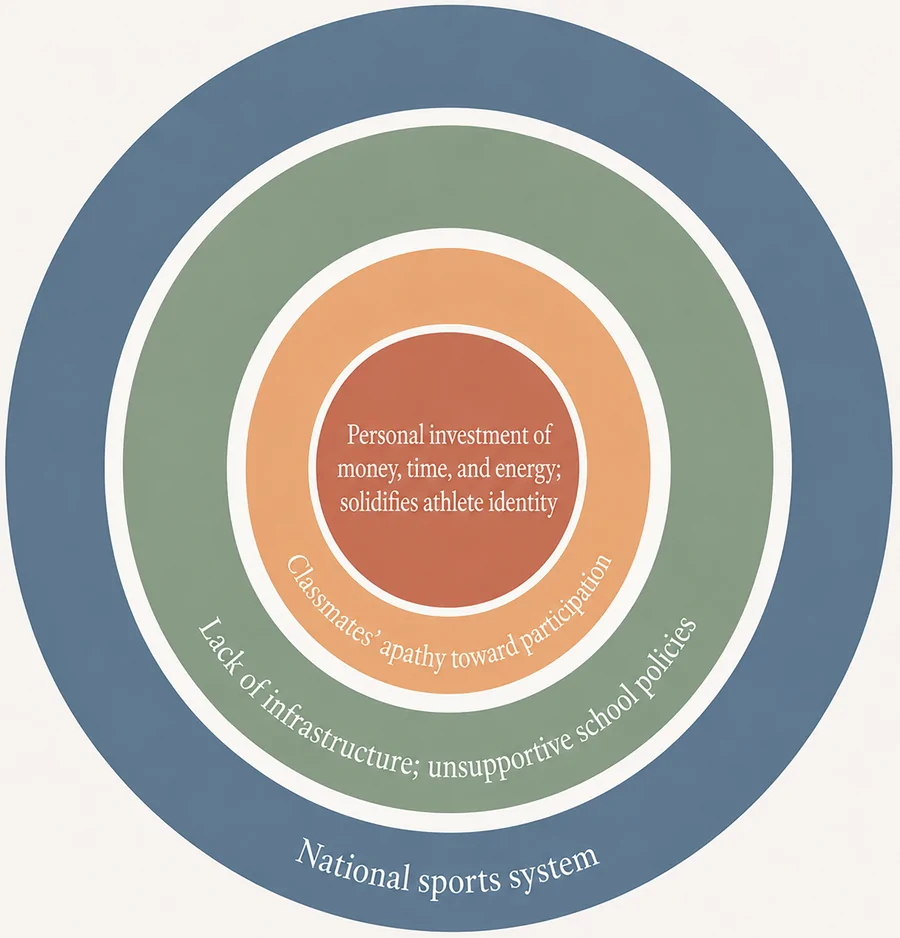
Social-ecological mechanism of athlete-identity centralization.

Before I truly stepped onto the field, my relationship with American football went through a prolonged phase of being “ignited but unable to be realized.” The education system's regulation of time and the marginalization of campus sports kept this interest at the level of imagination for a long period (Figure 2). Once the university finally provided discretionary time and space for exploration, this belated passion did not slow down. Instead, within a participation environment characterized by high costs and scarce resources, and accompanied by stigma and indifference, it quickly “compressed and nucleated” (Figure 3). It was precisely within this entanglement of structural resistance and individual investment that football shifted from interest to commitment. The athlete identity rose from a life option to the center of my self-concept, and began to spill over into academic and career aspirations, shaping a dual-track self as an “athlete–scholar.”.

#### 4.1.1 From fascination to the field

My engagement with American football can be traced back to watching the documentary *Hard Knocks* in 2016. Although I developed a strong interest at the time, I was unable to start practicing before university. This “disconnect between interest and practice” is not exceptional, but reflects a common condition among many Chinese students.

At the macro level, the highly competitive rules of the National College Entrance Examination (109) cascade into meso-level school management and present a highly structured form of “learning-centeredism.” Chinese high school students' schedules are strictly regulated: on average, they arrive at school at 7:00 and leave at 18:30, spending up to 11 hours on campus (110). Evenings often require compulsory self-study at school until 22:00, and Saturdays may also be used for makeup classes (111). My own high school life closely resembled this pattern. The rigid temporal structure deprived me of the physical time necessary to develop personal interests.

Although campus sports policies advocate for diversified development through sports (112), in practice, sports activities have long been marginalized. Even where limited physical education classes exist, their focus is often distorted into exam-oriented training for physical fitness test scores, rather than interest cultivation (113, 114).

These meso-level educational practices left me without a temporal and spatial arena for exploring the external environment and my own interests during middle and high school. The literature notes that limited early exploration often leads individuals to struggle with later career or major decisions (115), a difficulty I experienced directly. At the same time, Chinese public discourse widely promoted “instrumental rationality,” treating expected income and employment prospects—rather than personal interest—as the core indicators for choosing a major (116). Sport was weakened early on under exam-oriented schooling (117), and in discourse among teachers and relatives, and within school and family narratives, sport-related majors were often contrasted with “intelligence and academic ability,” framed as unserious, and burdened by stigma and traditional expectations regarding “proper” majors (118). Under this dominant narrative, I never dared to imagine pursuing a sport-related field that interested me. Instead, I complied and chose Software Engineering, which at the time appeared to offer the best salary prospects.

After entering university, my discretionary time increased sharply, and I gradually realized that I did not love my major. When individuals have not formed stable commitments and have long lacked arenas for repeated practice and self-verification, confusion about “who I am, what I want to become, and what I am doing every day” can erupt in a concentrated way (119). This realization activated my desire to reconstruct my identity and sense of commitment and pushed me to urgently search for an entry point, ultimately leading me to the field of American football.

#### 4.1.2 Sustained participation in China requires substantial personal effort

China's American football market is extremely weak, and at the meso level, the availability of clubs and teams is highly limited (120, 121). At first, I searched almost exhaustively across major social media platforms before I finally found a low-level team willing to accept a tryout.

However, this was only the beginning. The team's training base was 50 kilometers away. As a student at the time, the round-trip commute took three hours. To make it to practice, I needed to leave campus at 5:30 a.m. every Saturday to catch the first subway. Even with the most economical commuting option, a one-way trip costs 50 RMB. Equipment costs further intensified the burden: a secondhand, entry-level helmet alone cost 1,200 RMB. With a monthly living allowance of only 1,800 RMB, these expenses were a substantial financial strain. Monthly training costs consumed nearly one-fifth of my living budget and left my daily life constrained.

Ironically, even after traveling 50 kilometers and paying a high cost, the training environment remained poor. We could only train on an old soccer field. The artificial turf was too shallow, causing professional football cleats to slip frequently. When rehearsing red-zone tactics, we even had to carefully avoid the soccer goalposts still standing on the field.

This lack of infrastructure is also, in practice, a macro-level reflection of China's so-called whole-nation sports system. China's sports resource allocation follows a top-down logic, organized and coordinated by party-state and sports bureaucracies rather than by market forces. The Olympics function as a comprehensive carrier of national power narratives. Under this system, resource distribution is strongly shaped by bureaucratic performance assessments oriented toward Olympic gold medals (45, 122, 123). Whether a sport is included in the Olympic system can determine a sharp divide in its survival conditions, with the contrast in skateboarding before and after Olympic inclusion offering a clear example (124). Conversely, American football, as a non-Olympic sport, is institutionally marginalized: it lacks a standing governance unit and faces a vacuum in funding, talent development, local government investment, and media exposure (45, 46, 125, 126). Few local authorities are willing to allocate resources to build relevant infrastructure at the meso level.

Because away games often require athletes to self-fund travel and venue costs, I attempted to establish a campus club to obtain official subsidies, but ultimately failed. At the meso level, American football is not within the support systems of universities or sports bureaus, and cannot access reimbursements or subsidies in the way that prioritized sports such as soccer and basketball can (127).

Beyond institutional barriers, there were interpersonal influences. Although I could understand why some classmates hesitated due to economic barriers, I more often encountered indifference. Even at university, many classmates maintained a utilitarian approach to sports and were unwilling to participate in activities without course credits or visible returns (128). They could not understand investing in something without an explicit payoff. Stereotypes rooted in local social impressions (120, 121) also shaped reactions: some dismissed the sport as barbaric violence, and even questioned, “If it is fighting, why carry a ball?”.

#### 4.1.3 Becoming an athlete-scholar: identity and spillover across life domains

Ordinarily, an environment marked by scarce resources, interpersonal indifference, and institutional exclusion would undermine motivation (129). However, when individuals invest against difficulty and persist successfully, the self-identification connected to that behavior can strengthen over time (130). A strong athlete identity provided a deep source of drive. It helped me overcome fatigue and setbacks, strengthened my sense of self-worth (131, 132), and further produced a spillover effect, carrying values and ways of acting from sports into other life settings (133).

I began staying up late to watch almost all professional sports events, including F1, MLB, and NHL. By 2022, as my aspiration toward this domain deepened, I grew increasingly curious about knowledge in sports. This dispelled my stereotypes about sports major students and encouraged me—despite not being strong in exam-oriented learning at the time—to take the graduate entrance examination. Because I could find joy in nearly any sports-related setting, I believed the same could be true for studying and working. Not only did I succeed in the competitive entrance examination, but I also obtained an internship at NFL China that same year through my deep understanding of professional sports, opening a dual-track path that combined academics, career, and athlete identity.

In the academic arena, I performed well and met my teachers' high expectations. Chinese scholars and commentators commonly argue that there is a “more pervasive and more severe” gap between research and practice in Chinese sports (134). My unique experience in athletic practice and my professional league work background enabled me to ask questions with concrete, embodied understanding and depth. Recognition from my grades and teachers strengthened my self-efficacy (135). As systematic knowledge gave me tools to interpret a field that fascinated me, I began to view academia as a structured life plan and formed a firm goal of pursuing a doctoral degree.

My athletic career also reached a breakthrough in the first year of my master's program. That year, I formally joined the CNFL (China National Football League). During that summer, I trained intensively for almost the entire period under the defensive unit and strength coach, Richard (pseudonym). This high-intensity shared investment allowed me to clearly sense a transformation in my physical capacity, and improved fitness in turn gave me substantial competitive confidence (136). In my debut as the starting safety (Safety), I recorded strong statistics: three pass breakups and four solo tackles.

However, this progress also brought additional psychological weight. As training frequency increased, my relationship with Richard reflected findings from coach–athlete relationship research: frequent interaction can elevate an abstract authority relationship into a highly valued, partner-like bond (137). In addition, the norm of reciprocity suggests that when individuals receive wholehearted investment and cultivation, they often develop a strong responsibility to “repay” it with outstanding performance on the field (138). Richard's trust and expectations were both my on-field motivation and an invisible contract: I could not fail this investment.

### 4.2 The emergence of contradiction and a broken promise

The contradiction became apparent for the first time: I began to realize that my identity as an athlete was starting to undermine my academic identity, and my supervisor's reminder brought this tension into the open. Role exit theory would treat this moment as the “doubt—seeking alternatives” stage. Here, I was repeatedly pulled in different directions by sunk costs, anticipated regret, anxiety about “bodily time,” the scarcity of a starting position, and reciprocal obligations to repay others' investment. Each rational judgment that I “should leave” was rewritten by another force—an affective pull and a set of structural pressures insisting that “it could not end yet.” I remained on the field, trapped in a state of “knowing I should withdraw, yet being unable to withdraw.”

The penultimate regular-season game against the Shanghai Eagles (pseudonym) was a “win-or-die” match that would decide whether we could secure a playoff berth. In the fourth quarter, with only seconds left, the outcome hinged on the opponent's final drive. As the left cornerback (Cornerback), I locked onto the wide receiver. Even now, the number on that jersey is still carved into my mind.

I exploded out of my stance and dropped into my backpedal. At the instant he planted and broke inside, I lost half a step. Their quarterback saw the brief throwing window and released the ball immediately. However, I turned my hips and accelerated, forcing myself back into position, extending my arm to disrupt—the pass was broken up. I felt no joy because fear still consumed me: they still had one last chance.

On the final down, they emptied the backfield. As the ball was snapped, I ran step-for-step with the receiver into the end zone. The moment he looked up, I knew the ball was coming again. This time, almost by instinct, I reached out to swat it away. When the ball arrived at his chest, my hand was already threaded between his arms—I tried to punch it out before he completed control. However, the ball felt welded to him. It did not move.

We lost our balance and went down together. When I struggled to my feet, I saw the referee's arms raised—touchdown, good.

In that instant, hundreds of imagined solutions flashed through my mind, but none could be realized: time had expired, possession was gone, and the call could not be overturned. Facing the inevitable, final outcome, I felt an overwhelming helplessness. This had been the season I came closest to the playoffs, and it ended in the most brutal way.

At the same time, my stubborn overinvestment in my athlete identity began to take a toll on my other identity. After training, extreme exhaustion made me unwilling to do any further cognitive work.

“Zichen, I think you may be spending too much time on football. You still need to put your mind back into your studies.”

During a group meeting, Professor Wang looked at my academic progress—which should have been stronger—and delivered a serious warning. “Okay, Professor, I understand. After this season, I will retire.” That was the promise I made.

Behind that promise was a structural dilemma faced by an “athlete with academic ambition.” Research has noted that many athletes enter university with idealized academic goals, but as the intensity of their competitions increases, they often grow increasingly alienated from their academic work (139). In China, this is even more challenging for students aiming for doctoral admission: excellent coursework is far from enough (140). We must also accumulate performance-based academic capital through projects and publications (141). Academic pressure alone is sufficient to make many students anxious (142).

Rationally, I knew Professor Wang was right—but I broke my promise. I could not accept ending my athletic career there. This refusal first came from the “sunk cost effect”: I had poured too much time, energy, and money into football, which made it hard to let go, although further investment might not serve my future academic goals (143). I did not want to admit that past effort had been “wasted.” I wanted to leave a bigger mark on a national stage, such as the CNFL, and to write a more satisfying ending. Self-esteem at retirement is closely tied to the quality of one's athletic career (61). When goals remain unfulfilled, retirement is easily experienced as “ending in regret,” thereby damaging self-worth and increasing the risk of post-retirement identity confusion (144).

Second, another reason why my stubborn breach of promise appeared shortsighted in the face of my academic prospects was a misalignment in how I perceived “bodily time” and “academic time.”

“If there is still a chance to build my body into a Ferrari race car, this is my golden age. When I’m older, if I work hard, maybe I can still be a mellow Mercedes—but I will never reach that former ceiling again.”

What I called a golden age was, in essence, a subjective perception of time. When individuals view a period as a fleeting, irreversible window of opportunity, they tend to invest their energy in goals that feel most emotionally meaningful to them in the present (145). In China, a master's program lasts three years. This creates an illusion that “there is still time” academically: doctoral admission is three years later, and academic training has a long payoff cycle. By contrast, time and opportunity on the field vanish quickly. Thus, the Ferrari became my “possible selves”—people use a concrete image of “what kind of person I want to become” to organize goals and action (146).

In the end, I returned to the field behind Professor Wang's back. I had to work harder to keep that taut thread between academics and competition from snapping. As academic tasks grew heavier, trying to maintain athletic performance while also doing research often left me drained (58).

### 4.3 Lights out: a compound turning point

**Figure 4:**
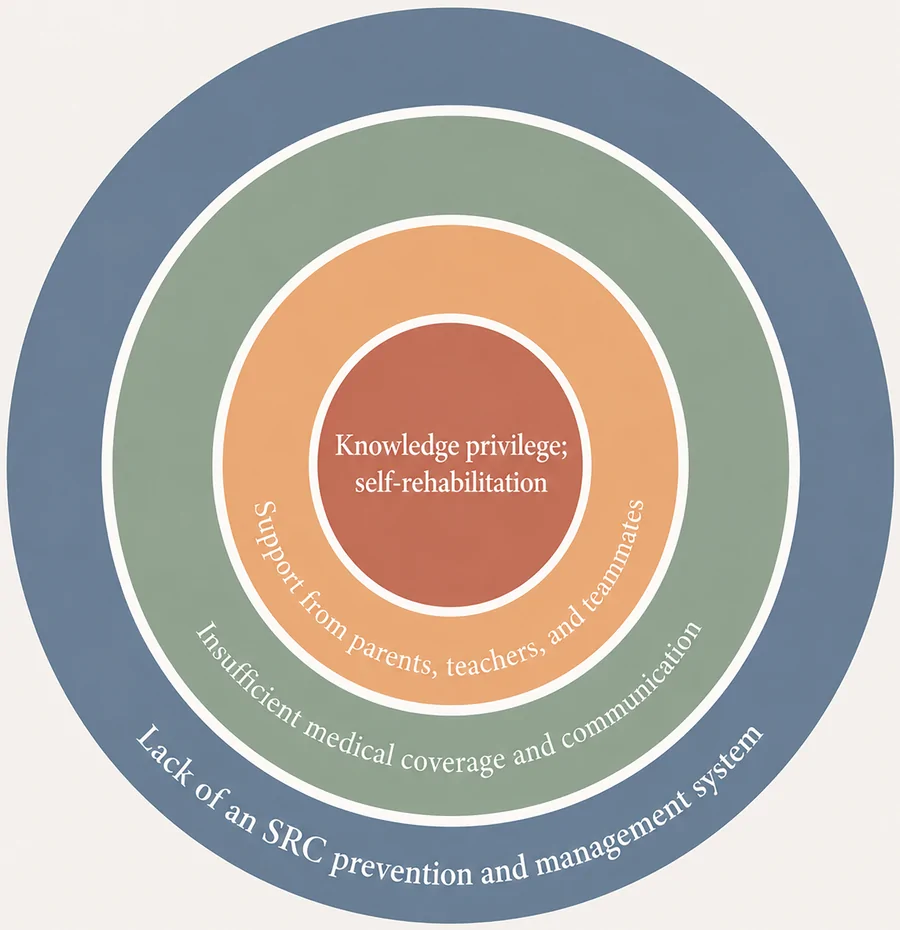
Social-ecological conditions shaping self-managed SRC recovery.

‘If earlier struggles remained at the level of “whether I am willing to exit,” after the concussion, the question became irreversibly “whether I can still afford the cost of staying.” The turning point in this stage was not a single moment of insight, but a chain of interlocking events: sudden amnesia and fear forced me to reassess bodily risk; immediate support from teammates, my supervisor, and my family temporarily stabilized my fragile emotions, but also made the severity of my situation clearer; the absence of medical communication and institutional protection also forced me to take responsibility for my recovery and decision-making (Figure 4). “Returning to play” was no longer a choice driven by love, but an adventure continually shoved forward by cultural norms and organizational competition logic. In the tug-of-war among the tough-guy narrative, the scarcity of starting roles, and self-deception, I increasingly realized that it was not simply that I “could not let go.” Rather, a highly centralized athlete identity had bound my meaning and dignity—until I discovered that fear had been permanently written into the hesitation before every collision, and that the athlete who could offer himself without reservation was disappearing. It was along this linked path—from injury, to doubt, to suppression, to admitting limits—that “exit” finally shifted from an option requiring repeated discussion to a reality that had to be faced.

#### 4.3.1 Waking up in the car: concussion, teammates, a good mentor, and family

In the 2024 preseason, we played away against the Jiaxing Hornets (pseudonym). For me, it should have been an ordinary red-zone defensive snap. I was already calculating how to respond with a vicious hit. After recognizing the opponent's run concept, I targeted the gap torn open by the offensive line and sprinted in at full speed.

Then, my memory stopped.

When I woke up again, I found myself sitting in my teammate Dart's (pseudonym) car. It felt like I had just taken a nap, but everything around me was strangely misaligned. “How did I take off my gear? How did I clean myself up?” I asked Dart, who was driving, full of suspicion: “Aren't we still supposed to play? Is it already over?”

“Neil (my name on the team), you must have a concussion. We’re taking you to a hospital in Hangzhou.” Dart paused. “Do you really not remember anything?”

“What? Who hit me?”

Because Dart was not on the field at the time, he could not answer. As I lowered my head to take out my phone to ask in the team chat, severe stiffness and pain in my neck snapped me back into reality—“Oh my god, did I really get a concussion?”.

The word “Concussion” had once felt distant to me. Compared with physical pain, a stronger fear surged up now: had I been knocked out in an extremely stupid way? If I had been knocked out because my technique broke down, that would be humiliating.

At that moment, my phone lit up:

“You made a beautiful tackle. Your pad level was very low, and the running back was hit so hard he flipped in the air. However, you were wobbling afterward—you must have had a concussion. We lost, but your defense was excellent.” [Diggs (pseudonym)]

“Neil, are you okay? I was scared to death—that tackle was beautiful! Rest well. We’re waiting for you to come back.” [James (pseudonym)]

Reading these messages, I felt a wave of relief. My taut nerves loosened, and I fell asleep again.

A sports injury is not only a physiological trauma; it is also a complex stress event. The acute phase (Injury Onset), the initial period immediately after injury, is a critical psychological window. During this period, athletes are often highly sensitive and unstable. They rapidly appraise their situation cognitively and closely monitor external cues (138).

In this micro-level arena of interpersonal interaction, teammates are often the first “proximal” social actors present and the first to interact with an injured person. Their support is therefore especially important (5). Looking back, teammate support during this experience primarily operated through two dimensions.

The first is instrumental support, that is, practical help and care. Dart driving me to seek medical care directly reduced real-world barriers, enabling me—physically vulnerable—to obtain treatment and examination in time. It made a chaotic emergency feel “controllable” and “manageable” (201–203). Being accompanied to medical care itself buffered stress, lowered panic and helplessness, and gave me emotional stability and a sense of safety. By contrast, when injured persons are left isolated, strong impatience and fear can easily emerge (129). I can hardly imagine the abyss into which the situation might have slid if, while mentally unclear, I had also needed to handle medical logistics alone.

Second is esteem support, that is, protecting the injured person's self-esteem and self-worth through words and actions. As friends closest to the competitive context, James's and Diggs's comments quickly dissolved my anxiety and shame about “possibly being injured in a stupid way.” Research indicates that such internal recognition helps reduce negative emotions in injured athletes and helps maintain positive self-evaluations, such as self-esteem and confidence (5, 147).

This aligns with a robust finding in social support research: support from “similar others” is often perceived as more “on point” and more effective. Teammates are “similar others” who share my role and situational knowledge (they understand the game and were there). Their support, therefore, carried higher credibility and a stronger basis for empathy, providing the most reliable coping model (148, 149). It was precisely this persuasive support grounded in shared experience that helped me move through the most chaotic hours after the injury.

“Zichen, are you okay? You’re going to Riverside Hospital (pseudonym), right? Your parents are extremely worried—remember to reply.”

When I woke up again, before I fully understood what was happening, I received a call from Professor Wang. “What? When did I tell Professor Wang? And my parents?” I turned to Dart in confusion: “Hey, do I have a concussion? Who packed my things? Why am I in your car? Where are we going?” Dart looked at me, amused and curious: “Neil, relax—you’ve asked those questions eight hundred times.”.

My memory was akin to a string of beads with a broken thread. I could not remember when I had last contacted my parents. Later, I learned that, while I repeatedly slipped into sleep, my mother had already boarded a bus from 230 kilometers away. My supervisor, meanwhile, had been at a faculty social dinner with foreign guests. After hearing that I was injured, he immediately excused himself from the dean and the guests and prepared to rush to the hospital in Hangzhou.

This reminded me of something. “Teacher, did I submit the final assignment for Sports Law? If I didn't, please forgive me for submitting it a few days late—I got a concussion in an American football game.” I quickly sent a message to the course instructor and received a reply almost immediately: “It's okay. You rest first. Submit it after you recover. Don't worry.”.

At the hospital, only after handing me over to Professor Wang did my teammates finally leave at ease. Walking out of the hospital, Professor Wang asked, “You must be hungry. Want to eat something?” I was still pretending to be tough: “Sure. Maybe the headache is just low blood sugar.” After devouring some fried chicken, Professor Wang walked me back to my apartment and said, “Your mother is already on her way. Rest well and wait for her.”

Throughout the process, he did not ask a single question, such as “Why are you still playing football?” This tacit “no interrogation” felt as if he understood that what I least needed at that moment was a lecture.

When my mother pushed open the apartment door, I dropped all my defenses. I confessed my fear: as a student, I understood how delicate the brain is, and I also feared that they would blame me or force me into the decision to “leave the team” before I was ready. However, she said nothing. She simply listened, poured me a glass of water, and stayed quiet. The next day, she accompanied me to a bakery as usual, chatting about everyday life. Before she left, I bought her a coffee and reminded her not to hesitate to take a taxi to the station. Even though she appeared calm and smiled in front of me, I could still see the fatigue from her long journey.

For student-athletes, social support from teachers and family after an injury is an indispensable resource (4, 150). Especially in the early stages, compared with informational support that instructs “what to do,” athletes often prioritize emotional support—being understood and comforted (151).

In this experience, my deepest fear was that my teacher and my mother would seize the moment to attack—with comments such as “I told you so,” or the question “Why are you still playing?” Psychosocially, this kind of response is essentially a combination of blame-oriented, moralizing negative social interactions and controlling communication. These interactions respond less to the injured person's “current needs” than to attribution, judgment, and pressure (152, 153), and can easily intensify athletes' anxiety and depression (154).

However, my teacher and my mother chose another approach: quiet listening and silent support.

Not only did my Sports Law instructor temporarily ease academic pressure through accommodation (155), but Professor Wang's immediate presence conveyed a strong sense of belonging. I was needed not only as an athlete; as an injured student, I was also valued. This understanding and acceptance greatly reduced my psychological strain in that moment (156).

For family, long-term bonds rooted in attachment can provide support that is more fundamental, more stable, and more dependable. This security allowed me to express vulnerability and regulate my emotions effectively (157, 158). My mother's arrival and her presence that day were a concrete embodiment of this type of support. It is also worth noting that family support is often a bidirectional stress event: parents are not only caregivers—they themselves experience worry, helplessness, and the pressure of negotiating with medical systems. Their experience is similarly shaped by the child's emotions and the external environment (159).

#### 4.3.2 Sent home without a map: self-managed recovery

“Two, four, six, eight…” I stared at the pill bottle in my palm and felt that the count was off. After counting, I realized that I had taken my vitamins four times that day—and each time, I forgot again almost immediately. “At least my memory is a bit better than yesterday, right?” I said to myself. However, when would I be considered “fully recovered”? I shook my head from side to side, trying to induce dizziness to assess my recovery status—a primitive and helpless form of self-testing.

My thoughts returned to the clinic the day before. It was a suffocating sense of misalignment. There was no expected SCAT cognitive assessment, not even a detailed consultation. The doctor's apathetic attitude left me at a loss. After a long CT scan and wait, the doctor looked at the screen, as if reading an inconsequential text message: “No blood clot. Probably a concussion. Go home and rest.”

“That's it?” My mind was already in chaos, and I could not organize the words to argue or press for details. I could only tilt my head, knit my brows, widen my eyes, and stare at him. That was how I was “dismissed” home—without any follow-up plan or management guidance.

Viewed at the macro level, my experience was not accidental. As of August 2025, China had not yet established an institutionalized sports-concussion law across provinces, as in the United States (160), nor did it have a unified management guideline covering grassroots to professional leagues as in Australia (161). China also lacked more finely grained protective policies, such as “return-to-learn” procedures (162) and staged “return-to-play” protocols (163). These policies should form a dense safety net—from “immediate removal,” to medical evaluation, graded progression, and educational support—aimed at minimizing secondary injury and reducing loss in academics and social functioning (26, 28).

Second, existing qualitative research shows that patients commonly want to be “informed” and to participate in their own health management, and that inadequate medical communication can easily generate stress through self-doubt (164). However, in the meso-level medical practice I experienced, this support was absent. The doctor provided no written information about the symptom spectrum or the expected recovery trajectory, did not explain how to increase activity in stages or how to avoid renewed head impacts (165, 166), did not specify follow-up points (22), or ask whether I needed medical documentation to secure rest entitlements (165).

This meso-level indifference made me feel dismissed and helpless. Fortunately, my academic background became a “lifeline.” Relying on my own health literacy, I attempted self-assessment according to the GRTP protocol (22): from getting out of bed and mopping the floor, to aerobic training, to shooting practice, and then to small-load strength training that avoided the Valsalva maneuver. I moved forward step by step through trial and error.

Even so, I remained confused and uneasy during recovery. The literature indicates that SRC recovery is strongly related to an individual's knowledge level and health literacy (166, 167). This forced me to reflect: if I found it difficult, then student-athletes in China's traditional centralized training systems, who may have a weaker ability to access knowledge and limited English proficiency (47, 168), would face an even deeper form of structural injustice due to the scarcity of meso-level support.

#### 4.3.3 Should I return to the field?

Three weeks later, I felt that I was “somewhat better,” but the feeling was fragile and uncertain.

In the gym, in front of the squat rack, I repeated silently, “OK, 130kg, that's it,” or “I won't add more.” Fear was an invisible wall. Even at the gym, it prevented me from returning to my previous training intensity. At the school track, my behavior became more absurd: I used a cheap foam pad that I bought online to build an upright obstacle, took a run-up, and knocked it down. I tried, comically, to use this crude simulation to rebuild bodily memory and confidence for tackling.

My phone vibrated. A message from a teammate appeared: “Hey Neil, how are you? Haven't seen you in training for the last two weeks. The regular season is about to start. When can you come back? We miss you.”

Staring at the screen, my fingers hovered over the keyboard. I replied: “I’m good, bro, don't worry.” But that was a lie. I was also afraid of losing my starting position. I wanted to write back: “Maybe I’ll go to the hospital for another checkup, and then I’ll be right back.”

“This is football, and in football you get hurt. Behave like a man.” Every head coach I had repeated this lesson. For a long time, I believed it. Football is hard, and I worshiped hardness. I disdained showing pain.

There is a one-inch blank line on my right arm tattoo where a teammate's helmet cut through skin and flesh during an Oklahoma Drill. A French coach once pointed at the wound and shouted, “Neil, you need to bandage it, it's bleeding.” I ignored him and replied in a somewhat boastful tone: “This is football, right?” It was only a flesh wound. It would heal. Although it hurt badly, I would not allow myself to show it. Similarly, when a much stronger offensive lineman crushed me and the chest impact nearly suffocated me, I only shook my head and forced out, “I'm good.”

In competitive sports, there appears to be an implicit contract: choosing to play means voluntarily accepting the possibility of injury. In this culture, pain, injury, and even long-term health loss are treated as “part of the game,” risk worth taking (32, 169, 170). In collision sports, such as football, “taking hits” and “standing up to impact” are not only techniques, but also symbols of competence and identity. Terms such as “Head strong” and “Pain zone” build a heroic narrative of playing through injury. Being able to absorb violent contact without going down is treated as a marker of toughness; in contrast, attending too much to pain can be labeled “weak” or “feminized” (30, 171, 172). These cultural norms often influence athletes toward biased symptom expression and return-to-play decisions during SRC recovery (173, 174).

In China, this pressure is further amplified by long-standing binary gender norms (175, 176). In public discourse and educational settings, “cultivating more masculine boys” is often framed as a national developmental task for the future. The image of the “physically strong, disciplined man” is naturalized as a template of personality and body for “strong-nation modernization,” and is embedded within the discourse of patriotic education (176–178). Under this pervasive social expectation, my psychological mechanism of refusing to reveal vulnerability in front of teammates—refusing to seem “fussy” or “nagging”—was further strengthened.

At the meso level of the team, a starting position is scarce. Organizational fairness can be cold. It will not reserve your place in case of injury or sympathy, or past contribution (179, 180). I knew that when attendance drops and performance becomes uncertain, replacement is inevitable. At the interpersonal level, the internal pressure not to disappoint teammates (181), intertwined with the anxiety of losing a starting spot, pushed me to downplay my symptoms and accelerate my return. This was a dangerous misjudgment (32). I fell into deep cognitive dissonance: rationally, I knew how fragile the brain is and that I might not be able to withstand another similar trauma; behaviorally, I was trying to deceive myself.

Although concussions are often invisible on routine imaging (22), the only comfort I felt I could obtain within the existing medical system was to undergo an MRI that looked more “precise” than a CT. I tried to use a scan report showing “no abnormality” to assuage my greatest fear about my body.

The new MRI result came out. “You look like you’ve recovered,” the doctor said, pointing at the images. I tried, in the brief moment after he finished speaking, to pour out information as quickly as possible to explain my condition. However, in the face of his indifferent expression, I felt powerless.

This powerlessness was not a mere overreaction on my part. China's healthcare system has long been shaped by inertia toward a paternalistic model, in which physicians occupy an absolute position of knowledge and power, while patient participation is structurally compressed (182). At the meso level of execution, this inequality becomes even more concrete: non-surgical recommendations are often delivered after diagnosis in a direct, simple, and seemingly unquestionable Procrustean manner (183). Moreover, constrained by extremely tight outpatient time and limited capacity for communication, physicians find it difficult to address an individual patient's hidden concerns. They rarely probe deeper needs through questions, rarely explain the dynamic trajectory of illness, and are even less able to provide preventive health education or behavioral risk counseling. Under such high-pressure conditions, full and equal communication becomes a luxury (184, 185).

“So you’re playing football?” The doctor glanced at the record, with an unsurprised tone of disapproval: “If you ask me, you should stop playing football. It’s far too dangerous.”

I knew that sentence too well. Joint inflammation, finger dislocation—no matter why I came here, the “medical advice” always converged on the same endpoint: not how to exercise more safely, but to stop playing sports altogether. I instinctively wanted to argue, but I swallowed my words. I furrowed my brow again, forced a bitter smile, and walked out of the clinic in silence.

#### 4.3.4 It is hard to let go

After returning home for the holidays, my mother discovered that I was still participating in high-intensity games. This was an unspoken “minefield” between us, but she eventually stepped on it and tried to persuade me to leave the field.

“Mom, football gave me everything. You should know: now that I can enter the sports field, whether in work or in academia, what underlies my achievements is football. Cutting it off is not that simple. You need to give me time to transition.”

Seeing the worry in her eyes, I looked strained and ended the conversation almost evasively: “That's it. Don't discuss it. It's not helping.”

It was not that I had never considered retirement. But deep down, I was terrified: if I stripped away the identity of “football player,” what would my movements mean when I walked into the gym again and grabbed that cold equipment? What would my strict dietary control be for? I feared a vast hollowing of meaning.

On the academic path, I had long taken pride in a kind of “dual privilege”—I was not only a scholar, but also a “warrior” who had bled on the field. This identity even fed an unconscious arrogance. As a model “excellent student,” I once bluntly criticized colleagues in front of my supervisor—those who sat in offices staring at screens and complaining that papers were hard to write: “If you haven't broken bones for sport, if you haven't bled, how can you do sports research well?”

“Football player”—this identity had been my hardest armor. In my most vulnerable moments—shivering at dawn to catch the first subway, living with financial constraints, swallowing blood under cold stares and heavy hits—it was this identity that reminded me with pride: you are different; you have desire; sport will repay your suffering.

However, when the crisis—the concussion—arrived violently and abruptly, the very bond that once had saved me instantly became a shackle I could not escape.

For individuals with dual identities as students and athletes, the stronger the athlete identity, the more difficult retirement and transition become (144). A strong athlete identity had been my deep driving force, giving me the resilience to overcome hardship (130–132). But when health considerations and academic futures collided, this overly rigid identity instead blocked rational decision-making and intensified resistance and anxiety about “not being able to let go.”

The concussion shattered, violently and suddenly, the delicate balance I had maintained between academics and sports. This kind of non-planned exit often makes it harder for athletes to accept (54, 186). I suddenly faced the fear of losing my sense of valued goals (187). Under the pull of athlete goals, years of high-intensity discipline had already trained my body and daily routines. Sudden stillness meant more than the suspension of physiological function; it was an ontological panic—this quiet was unfamiliar and suffocating (68, 188).

#### 4.3.5 The final down: it is time to leave

The reality was that, on an unforgiving field, I truly was no longer the same. I had slowed down.

In the past, I treated speed, ferocity, and total sacrifice as weapons. I held nothing back in every tackle. Collisions even excited me—they were a promise to my teammates, a hunger for victory, and the utmost respect for the sport.

“Maybe my tackle position is wrong.” On the practice field, I refused to accept that and tried every technical detail—adjusting the head-across tackle, optimizing the angle of the rugby tackle—in an attempt to draw a thin, illusory sense of safety by changing the way I collided. However, nothing changed.

Now, before every collision, a fatal hesitation flashed through my mind. After every tackle, a cold fear rose: fear that my brain would “power off” again, fear that the terrifying buzzing would return.

I realized that I could no longer offer this sport, my team, and myself my 100% fearless self. I could not accept facing them with a partial, diminished self.

In the first round of the playoffs, we faced the Shanghai Eagles again. Our special teams had three kicks blocked. Attempts on an onside kick failed one after another. The situation collapsed rapidly. As with the Denver Broncos in the 2014 Super Bowl, we quickly became sitting ducks, and the suspense drained from the game.

During the final defensive series, I still stood at left cornerback. However, unlike a year ago, what filled me now was relief instead of tension. Looking at the scoreboard, I realized that perhaps this season was the time to draw the line. My football career flashed through my mind like a lantern show…

I still had my doctoral studies. I still had a body that was, for now, healthy. And I still had a mother who no longer needed to worry herself sick over me. After all, according to the original plan, I should have left in 2023. This extra year of playing had already been a gift.

A voice said to me, “Neil, this is your final down. Play it with everything you have.”

After the game, I submitted my request to leave the team. The team owner replied, “Neil, go pursue your dreams. I wish you great success in your studies.”

### 4.4 After the whistle: creating the ex-role

When I finally submitted my request to leave the team, role exit did not end with “saying goodbye to the field.” On the contrary, it entered a more concealed yet more enduring phase: I had to learn how to become a “former athlete.” The athlete identity did not disappear. It persisted in residual form—bringing with it both the temptation to return and the fear of a hollowed-out meaning, while also providing a distinctive form of capital for entering the field, understanding athletes, and building trust. The core task here was not simply “letting go,” but reconfiguration: removing the old identity from its dominant position in everyday life, while re-embedding it, in a new form, into my academic and life trajectory.

“We take care of each other.” In the year after my retirement, after an Asian baseball championship, I shook hands with a Canadian-Palestinian player as if we were brothers as we said goodbye. Only days earlier, he had suffered a shoulder dislocation during the tournament. Although my role in that match was merely as a media officer, I became the person in the entire venue who understood the athletes best.

I accompanied these English-speaking athletes to a local clinic for rehabilitation, translated for them, and even showed them how to watch the NFL on China's internet. Doing work outside my formal duties brought me a long-missed sense of joy—because, as an athlete myself, I knew what one needs the most when injured in a foreign place.

This familiar sense of connection also continued in my fieldwork. “How do you know?” youth athletes often asked in surprise when my questions hit close to home, touching on their sporting lives and private inner worlds.

I simply smiled: “Because I used to be an athlete too.”

Gradually, I became known as a researcher who “understands athletes.” I had survived inside the most brutal meat grinder of competition. My identity as an athlete did not dissolve with retirement; instead, it continued and was reshaped within academic research.

I began focusing on athletes who survive within structurally unequal environments and thinking critically about the double-edged influence of sports on personal development. In 2025, my first SSCI article was successfully published. This gave me an additional asset in my doctoral trajectory and allowed me to rediscover a long-lost sense of purpose. The academic path that I once had to maintain with difficulty on top of high-intensity training and competition now offered another exit, enabling me to rebuild a self I could again be proud of.

Previously, what I felt most strongly was the simultaneous and persistent pressure of academics and sports, a dilemma shared by many student-athletes (189, 190). When “athlete identity” becomes overly dominant within the self-concept, students' academic achievement goals and academic engagement can be systematically suppressed, thereby weakening academic output and subsequent academic pathways (191).

However, sports and academics are not only in conflict. They can also complement each other and provide another pathway after retirement (192). When sustained, education contributes significantly to the quality of post-sport transitions (193). As research indicates, athletes who retire with higher educational attainment and stronger academic resources often have better employment outcomes and higher life satisfaction (61, 194). The academic capital that I had unintentionally preserved became a buffer and a bridge. I consciously reworked the “role residual” of my former athlete identity into a new identity (18, 195). This became a unique advantage in building trust in new research contexts, providing cultural capital for entering the field (196). It enabled me to understand the data more authentically and to facilitate deeper accounts and resonance during interviews (204, 205). Engaging in dialogue with “similar others” essentially extends the dividends of a shared identity (206), producing experiences of being understood and accepted, thereby enhancing positive affect and subjective well-being (207).

“Neil, eat more meat.” “Neil, is there still a chance you’ll come back? How about joining the Shanghai team?”

In a hotpot restaurant, steam rose into the air. Several teammates from the defensive unit came to campus for training and took the opportunity to have a meal together. Hearing them talk about the team's recent situation, truthfully, I missed the brotherhood in the locker room more than anything. I missed the adrenaline of game day and the ecstasy of fending off opponents again and again. To say I did not want it would be dishonest.

However, I knew the story of Athlete Neil had ended. I was no longer the defensive back wearing the green No. 20 jersey. I was an ordinary person named Zichen.

I love everything about this sport. Precisely because of that, I made a difficult, yet most important decision: to stop myself from sinking into it, and to turn toward a future full of uncertainty—perhaps even one that looked dark. Fortunately, I remained physically strong, healthy, and intact. Now, I had begun a new chapter.

## 5 Conclusion

Using autoethnography, this article offers an in-depth analysis of how a Chinese, non-system student-athlete, after experiencing a sport-related concussion (SRC), completed a full process of “role exit” under the simultaneous pressures and supports of multiple social ecological systems. This analysis shows that SRC is not a singular biomedical event. Rather, it is a stress trigger that “speaks” across multiple social levels at once: it brings back anxieties, shame, responsibility, and institutional uncertainty that had previously been suppressed or rationalized, and it continues to shape how individuals judge risk, meaning, and the future during recovery and decision-making. For this reason, the decision of “whether to continue” is never purely rational or determined only by symptom severity. Rather, it is an ethical life decision jointly influenced by identity, relationships, organizational logic, medical communication, and macro-level institutions.

Where does anxiety come from, how does it obstruct rational decision-making, and why does it re-emerge when SRC occurs?

At the individual level, the core of anxiety in this case arose from the rapid centralization of athlete identity and the temporal perception of an “irreversible window”: sunk costs, anticipated regret over an unsatisfactory ending, obsession with peak bodily performance, and demanding self-expectations regarding academic prospects all coexist. This produced prolonged cognitive dissonance between “rationally I should withdraw” and “emotionally I cannot withdraw.” After the SRC occurred, amnesia and fear amplified catastrophic thinking. Meanwhile, “normal” imaging generated a new uncertainty, prompting the athlete to treat “medical confirmation” as a substitute for soothing fear, thereby reinforcing a cycle of repeated medical visits—self-doubt—reassurance seeking.

At the interpersonal level, the norm of reciprocity and team expectations within coach–teammate relationships moralized “return”: Author ZZ was not only playing for himself, but also for the investment and trust that he “could not betray.” At the same time, masculinity and pain culture, through daily admonitions and locker-room norms, continually define what it means to be a “qualified football man,” making symptom disclosure and cautious rest more easily experienced as “weakness,” “a burden,” or “losing one's spot.” These cultural scripts did not fade after injury. Instead, they intersected with organizational competition logic under starting-position scarcity, converting into stronger impulses to return and deeper self-deception.

At the meso level, the absence of support from healthcare providers and schools/organizations magnified anxiety: the lack of systematic evaluation, the absence of clear follow-up and graded recovery guidance, and the absence of executable “return-to-learn/return-to-play” pathways forced recovery to become privatized and experiential. Individuals must rely on personal health literacy to fill institutional gaps. However, this “self-managed recovery” itself pushes risk and responsibility back onto the individual, making rational decision-making even more difficult.

At the macro level, structural conditions—including the low degree of public problematization and institutionalization of SRC in China, the long-term marginalization of non-Olympic sports, and the tendency toward paternalistic medical communication—jointly constitute “invisible environmental pressure.” These may not appear as direct commands, but they continuously shape interpretive frames and behavioral boundaries through resource accessibility, information structures, social evaluation, and opportunity constraints. Thus, what re-emerges when an SRC occurs is not only pain or dizziness, but the multi-directional pulling forces of the entire social ecological system surrounding the question of “whether to continue.”

During recovery and role exit, what factors buffered and supported the process?

Buffering forces also came from multiple levels, but their mechanisms were more strongly characterized by “proximal processes”: they often appeared at key moments in the form of interaction, companionship, and permission.

At the interpersonal level, teammates' instrumental support (e.g., driving to the hospital, caregiving) and esteem support (e.g., affirming performance, protecting self-worth) provided psychological anchors of “controllability” and “continued recognition,” substantially reducing shame and panic. When author ZZ feared moralizing blame the most, his supervisor and course instructor instead offered emotional support by “not interrogating” him and through academic flexibility, temporarily reducing external pressure from dual-track identity conflict. Family support provided a more stable sense of security, allowing him to express vulnerability and facilitating emotion regulation and meaning reconstruction.

At the individual level, health literacy and academic capital were key resources for self-managed recovery: the ability to search for, understand, and attempt graded recovery protocols preserved a degree of agency in the absence of institutions. More importantly, education and a research pathway provided a concrete anchor for post-exit identity reconfiguration, enabling the “role residual” of athlete identity to be transformed into cultural capital in research and an advantage for field access—supporting a shift from “meaning void” to “meaning transfer.”

At the meso and macro levels, although the author's lived-in experience was more often one of absence and roughness, this very absence highlighted the potential value of institutional support. If schools, healthcare systems, and league organizations could provide clear procedures, responsibilities, and resources (e.g., academic adjustments, medical documentation, graded return protocols, and risk education), then fear would be less dependent on guesswork, and decisions would be more likely to be discussable, negotiable, and actionable.

This article does not aim to use a single case to “represent everyone.” Rather, through embodied narrative and a social ecological framework, it addresses seemingly private questions—“Why did I persist?” “Why was I afraid?” “Why was it hard to let go?” “How did I become a former athlete?”—as public issue that are open to institutional, cultural, and practical intervention. At the same time, it reveals the invisible impacts of “invisible injury” among athletes (e.g., shame, hesitation, collapse, and reconstruction of meaning), thereby offering an experiential supplement to the scarce lived-in experience perspective within Chinese SRC research and providing context-sensitive conceptual clues for future qualitative and mixed-methods research.

As an autoethnography, this study is inherently constrained by a single subject and contextual specificity. Future work could include more qualitative and mixed-methods studies with student-athletes from different regions in China, different sports, genders, and positions within and outside institutional systems. These studies could also evaluate the feasibility of implementing coordinated school–healthcare “return-to-learn” and “return-to-play” processes in China, and further test how sociocultural norms concretely shape symptom disclosure and the quality of exit transitions.

While individual choices may help “me” avoid the next collision, they cannot resolve the broader structural dilemmas faced by larger groups. China urgently needs to establish cross-sector mechanisms linking healthcare, education, and sports, develop a localized SRC prevention and support system, and reshape a more inclusive sports culture. Only then can future student-athletes, when facing injury, rely not on luck or individual bravado, but on a stable safety net—one that allows them to pursue their competitive dreams while still preserving a healthy future. I have turned away from that grass field, but I hope my narrative can light—even faintly—a small lamp for those who are still on it. I also hope my story resonates with student-athletes in similar circumstances: sometimes, accepting a draw before the position collapses requires more courage than playing on.

## Data Availability

The original contributions presented in the study are included in the article/Supplementary Material, further inquiries can be directed to the corresponding author.
